# Knowledge and preventive actions toward COVID-19, vaccination intent, and health literacy among educators in Japan: An online survey

**DOI:** 10.1371/journal.pone.0257552

**Published:** 2021-09-20

**Authors:** Yasue Fukuda, Shuji Ando, Koji Fukuda

**Affiliations:** 1 Faculty of Pharmaceutical Sciences, Suzuka University of Medical Science, Suzuka-city, Mie, Japan; 2 Department of Information and Computer Technology, Tokyo University of Science, Tokyo, Japan; 3 Faculty of Political Science and Economics, Waseda University, Shinjuku-ku, Tokyo, Japan; International Medical University, MALAYSIA

## Abstract

Countermeasures against the spread of COVID-19 have become an urgent issue in educational settings, where many group activities are necessary. Educators are key to preventing the spread of COVID-19 in educational settings. Infection prevention behavior requires comprehensive and complex measures such as self-restraint. disinfection care, hand washing, wearing masks and recommendation and implementation of vaccination. Improvement in the knowledge, skills, and preventive actions of educators vis-à-vis COVID-19 could allow for the continued provision of educational services while ensuring safety in educational settings. Therefore, the objective of this study was to explore the knowledge and preventive actions of educators regarding COVID-19 and vaccination awareness to provide appropriate support for educators. The study used data collected from 1,000 Japanese educators in January 2021 when the third wave of viral infections spread. Online surveys and multivariate linear regression analysis were used to determine age and whether respondents were being cared for by a doctor. We investigated the effects of factors on educators’ willingness to be vaccinated and changes in their behavior. This study found that factors such as age, gender, whether a respondent was under a physician’s care, and health literacy, affected the willingness of educators to receive vaccinations and engage in preventive actions. The study also suggests that the reliability of national government public relations efforts is lower than the reliability of local government public relations and that of information from family physicians, pharmacies, and mass media. It is therefore necessary to reexamine how information is disseminated by the national government and to increase the degree of trust in that information among the public. The findings of the study also revealed the importance of improving the provision of appropriate information and health literacy for the behavior of educators, not only during the initial outbreak, but also during the subsequent period of pandemic life.

## Introduction

Outbreaks of novel viral infections, such as COVID-19, has shed light on situations in which the public has no choice but to manage and suppress the spread of infection by restraining and altering their own behaviors, given the unavailability of cures or treatments and uncertain methods of prevention [1–4]. The competition to develop vaccines as a means of managing the spread of COVID-19 has accelerated, with vaccines being adopted for practical use based on a rapid, but atypical approval process. As of February 2021, vaccinations have been initiated in several countries around the world [5–10]. To control the spread of infections, it is important to facilitate vaccinations for people without symptoms, the research and development of therapeutic agents, and the examination of treatment methods for those affected by the virus. Yet, the most effective way of dealing with the outbreak of COVID-19 currently is for people to control their behavior and transform their lifestyles, like using face masks liberally, staying home as much as possible etc.

When the spread of the infections began at the end of February 2020, school closures were implemented in Japan. Elementary schools, junior high schools, high schools, and universities nationwide were closed all at once from March to early May [11]. Around the same time, schools were closed in the United States and the United Kingdom [12, 13]. Online on-demand lectures were introduced in a few private schools and many universities [14, 15]. However, the Internet learning environment is not an optimal one for the students, hence public opinion is increasingly in favor of reopening schools. Since May, face-to-face lessons have resumed in many schools in Japan [16]. In other countries as well, several researchers have pointed out that mental health problems among students would have increased if schools were to remain closed and schooling from home [17, 18]. Unfortunately, while schools have reopened in many countries around the world, some of these schools have also formed COVID-19 clusters [19, 20].

With the spread of COVID-19, it has become an urgent issue to balance infectious disease measures and continue educational activities, not only in medical settings, but also in educational settings, where many group activities are necessary [21]. Educators have a responsibility to protect themselves and students from infectious diseases, thus they should be a model for student behavior. Educational institutions, the national government, and administrative agencies must provide methods enabling educators to manage the COVID-19 situation, to support and strengthen educational activities, and to support educators in reducing their psychological and physical stress. Enhancing the knowledge and attitudes about COVID-19 among educators would ensure safety at the learning institutions. While studies on the knowledge, attitudes, and preventive behavior of college students regarding the COVID-19 have been reported [22–24], few studies have been conducted on educators’ attitudes, preventive behaviors, sources of information, and confidence. The purpose of this study is thus to explore educators’ knowledge and attitudes about COVID-19, their awareness of infection prevention, and their overall management of the COVID-19 situation.

## Materials and methods

### Survey design and participants

An internet survey was conducted during January 8–11, 2021, and both univariate and multivariate analyses were carried out on the questionnaire results from 1,000 respondents working at educational institutions throughout Japan. Survey items investigated in the study were demographic characteristics of educator attributes, such as age, gender, institution of employment, academic background, as well as their knowledge of COVID-19, preventive actions, vaccination intent, the awareness and reliability of medical information sources, and health literacy.

We derived a minimum sample size estimate of 385 based on a normal approximation of the binomial distribution with a finite population correction applied [25] (assuming an observed proportion of respondents selecting a specific response option of 50%). This incorporated educator population size, which according to the statistics provided by the Ministry of Education, Culture, Sports, Science and Technology, numbers around 1 million [26] with a 95% confidence level, and a margin of error of 5%. From a previous study, we elected to collect 1,000 questionnaires to improve the study’s validity [22, 27, 28].

### Selection criteria

Eligible study participants were men and women who are at least 20 years of age, are members of educational institutions in Japan, and those who expressed their intent to participate after receiving an explanation about the study on the Internet.

### Exclusion criteria

Participants with inconsistent registration details and responses were excluded from the study. In order to exclude participants who did not carefully read the questions with five multiple-choices, a trick question that asks the respondent to select a specific symbol was utilized. The responses from those who failed to select that symbol were invalidated and excluded.

Gender was evenly assigned to each age group, and a questionnaire was distributed to 6,247 people who registered their occupations as teachers. In total, 1,964 people agreed to participate, however, 326 participants whose responses in this study did not match their age, gender, and educational history at the time of registration were excluded. The number of complete responses was 1,175, of which, 151 participants did not respond to the trick question appropriately and were excluded. We also excluded data from 24 respondents who recorded significantly shorter response times compared to the other respondents.

In total, the results of the questionnaires from a total of 1,000 respondents with complete and consistent responses were analyzed in this study.

### Study items

The variables included in the study were as follows:

#### (1) Respondent demographic characteristics

Age group, gender, educational level, and order of employment.

#### (2) Knowledge regarding COVID-19

Knowledge regarding COVID-19, infectious diseases (5 multiple-choice questions) and knowledge of infection routes, bacteria, and viruses (5 multiple-choice questions)

Questionnaires from previous research regarding knowledge and based on WHO guidelines were modified by region, culture, and participant attributes [22–24, 3]. In Japanese educational settings, the need for hand washing and masks is obligatory in educations settings based on the guidelines of the Ministry of Education, Culture, Sports, Science and Technology [29, 30]. The knowledge part of the existing research is already commonly understood in Japanese educational institutions. Various symptoms have been reported for the symptoms, and individuals’ knowledge often overlaps with their attitude [22–24, 31–34]. Discussions were held between experts and researchers, and the questions were narrowed down to two.

The choices for questions regarding the transmission route were “sexually transmitted diseases,” “mainly droplet infections and contact infections,” “mosquito-borne,” and “due to food poisoning.” The choices for questions about bacteria and viruses were “viruses can grow on their own,” “bacteria cannot grow on their own,” “viruses are larger than bacteria,” and “viruses do not multiply in food.”

#### (3) Health literacy

Health literacy and infectious disease prevention education at school are indispensable for controlling illnesses [35]. In this study, the European Health Literacy Survey Questionnaire (HLS-EU-Q47) was used to measure the health literacy of teachers [36]. This HLS-EU is a measure of health that is aimed at promoting knowledge of infectious disease prevention and improving quality of life. The HLS-EU comprised 47 questions, divided into three areas, namely health management, disease prevention, and health promotion. It is an index that integrates the four information processing capabilities of access, understanding, evaluation, and application. This index was evaluated on a 4-point Likert scale with the following options: 1 = “very difficult,” 2 = “difficult,” 3 = “easy,” 4 = “very easy,” and 5 = “don’t know”. The “don’t know” option was treated as a missing value. [36–38] All scores were converted into a unified metric with a minimum of 0 and a maximum of 50, where 0 represented the “least possible” and 50 represented the “best possible” health literacy score. The HLS-EU has also been used by Denuwara to measure the health literacy of educators [39].

#### (4) Preventive actions

Behavioral self-restraint and proactive health behaviors (like disinfection and seeking dental/medical care) were scored to measure preventive actions adopted during the COVID-19 pandemic. Questions about preventive actions comprised 10 items such as self-restraint, disinfection, and consultation at a medical institution for physical condition management. These individual items were evaluated as 1 (yes) or 0 (no), and the total score was calculated, after which it was defined as preventive action.

#### (5) Vaccination intent

Vaccination intent, or the expression of intent to vaccinate, was measured based on timing The choices were as follows: 5 = “immediately after inoculation becomes possible,” 4 = “six months after inoculation becomes possible,” 3 = “one year after inoculation becomes possible,” 2 = “two years after inoculation becomes possible,” and 1 = “will not inoculate.” We also asked for a free statement as to why respondents choose to vaccinate at that time.

#### (6) Medical and health information sources and reliability of information

Multiple answers were established. Participants were asked what sources they used for health and medical information and to rate their reliability on the following 5-point Likert scale: 1 = “untrusted,” 2 = “somewhat unreliable,” 3 = “neither,” 4 = “somewhat reliable,” and 5 = “reliable.”

### Data analysis

We summarized demographic characteristics based on gender, age group, affiliated educational institution, education history, degree. Continuous variables are expressed as means ± standard error (SD).

Univariate and multivariable linear regression analyses were performed to identify factors related to health literacy, attitudes, and actions regarding COVID-19 prevention and willingness to undergo vaccination. SPSS Statistics version 26 (IBM NY, USA) was used for all statistical analysis, and the significance level was set at 5%.

### Ethical considerations

In this study, no information was obtained that could be used to identify any of the participants. Informed consent was obtained online. Research participants were required to read an explanation of the purpose and method of the research, a description of the research procedure, a description of the risks, information on the voluntariness of participation, how the information would be disclosed, and how the data would be handled. After they have been suitably informed, they had to check a box to agree or disagree. Those who agreed with the explanations proceeded to the questions. Respondents could withdraw their consent in the middle of an answer and cancel their participation. In such a case, that person’s information would be excluded from the analysis. The contact information of the principal investigator was specified as the contact information for this research. The collected information was completely anonymized, and no personal information was obtained. There are no conflicts of interest. Approval for this research was obtained in November 2020 from the Waseda University Research Ethics Committee (Approval number: 2020‐297).

## Results

### (1) Survey respondents’ demographic characteristics

Table 1 presents the demographic characteristics of the respondents. The respondents’ age groups were as follows: those in their 50s made up 40% of the total, those in their 60s made up 24.6%, those in their 40s made up 20.6%, those in their 30s made up 11.1%, and those in their 20s made up 2.9%. In descending order of employment are those working at high schools (29.2%), elementary schools (24.8%), universities (21.1%), junior high schools (18.3%), and junior college/vocational schools (6.6%).

**Table 1:** Participants’ demographic characteristics.

	Socio-demographic characteristics	N	%
Gender	Male	769	76.9
	Female	231	23.1
	Total	1000	100
Age	20s	29	2.9
	30s	111	11.1
	40s	206	20.6
	50s	408	40.8
	60s	246	24.6
	Total	1000	100
Employment	Elementary school	248	24.8
	Middle school	183	18.3
	High school	292	29.2
	Vocational school/junior college	66	6.6
	University	211	21.1
	Total	1000	100
Employment type	Full-time	811	81.1
	Fixed-term	65	6.5
	Part-time	124	12.4
	Total	1000	100
Educational level	Bachelor’s	651	67.5
	Master’s	163	16.9
	Doctorate	151	15.6
	Total	965	100

The total number of educators at elementary schools, junior high schools, high schools, junior colleges, universities, and graduate schools in Japan is 1,088,212 according to data from the Ministry of Science in 2020. According to that data, the percentage of women working in education was highest in elementary schools at 62%. However, the percentage of women decreased as the level of education increased, with 43% in junior high school, 32% in high school, and 25% in university [26]. We delivered questionnaires in consideration of the gender ratio, but not all respondents gave complete answers. In terms of respondents’ sex, 76.9% were male and 23.1% were female.

### (2) Knowledge of infectious diseases

Regarding knowledge of infectious diseases, there were two questions about infection routes and bacterial/viral infections. Altogether, 99.9% of respondents answered correctly regarding the infection route, “mainly droplet infection and contact infection” (respiratory droplets droplet contact infection), and 33.5% of respondents correctly answered that a “virus does not propagate in food.” The most popular answer choice for the difference between bacteria and viruses was “viruses can multiply on their own” at 46.2%.

### (3) Health literacy

The average total health literacy score for educators was 33.5 ± 7.61 (converted to a maximum 50-point scale). In Tables 2 and 3 below, health literacy is the dependent variable (objective variable), and age, gender, academic background, and highest educational attainment are the predictive variables. Table 2 shows the univariate analysis of health, and Table 3 shows the multivariate analysis of health literacy and predictive factors. An association was observed between health literacy and age, as well as highest educational attainment.

**Table 2:** Univariate linear regression analysis of health literacy, predictive factors.

	Non-standardized coefficient		P-value	B 95% confidence interval	
	B	Standard error		Lower bound	Upper bound
(Constant values)	32.138	0.542	0.000	31.074	33.202
Educational level	0.910	0.327	0.005**	0.269	1.551
(Constant values)	30.819	1.117	0.000	28.627	33.010
Age	0.569	0.231	0.014*	0.116	1.021

*p<0.05

**p<0.01.

**Table 3:** Multivariate linear regression analysis of health literacy, predictive factors.

	Non-standardized coefficient		P-value	B 95% confidence interval	
	B	Standard error		Lower bound	Upper bound
(Constant values)	29.123	1.246	0.000	26.678	31.568
Educational level	0.916	0.326	0.005**	0.277	1.555
Age	0.634	0.236	0.007**	0.171	1.097

**p<0.01.

### (4) Preventive actions adopted under the COVID-19 pandemic

Table 4 indicates behaviors and attitudes about COVID-19. Regarding self-restraint, 14.0% stockpiled daily necessities, 74.7% refrained from going out, 92.9% refrained from sightseeing and traveling to tourist spots, 95.9% refrained from get-togethers that involved eating/drinking, and 91.1% refrained from using amusement/entertainment facilities.

**Table 4:** Preventive practices at stage of COVID-19 third-wave.

	Preventive practices		N	%
Self-restraint of activities	Do you stockpile daily necessities?	Yes	148	14.8
	Do you refrain from sightseeing and traveling to tourist spots?	Yes	929	92.9
	Do you refrain from get-togethers that involve eating/drinking?	Yes	959	95.9
	Do you refrain from going out?	Yes	747	74.7
	Do you refrain from visiting amusement/entertainment facilities such as pachinko parlors?	Yes	911	91.1
Preventive actions	Do you routinely check your body temperature?	Yes	711	71.1
	Do you routinely disinfect doorknobs and cellphones?	Yes	440	44.0
	Do you work remotely and take care to maintain social distancing?	Yes	682	68.2
Holding off on health examinations	Do you hold off on being examined by a physician?	Yes	358	35.8
	Do you hold off on being examined by a dentist?	Yes	354	35.4

A total of 35.0% held back on medical appointments and 35.5% held back on dental appointments. In terms of activities such as measurement of body temperature, disinfection, and maintenance of social distancing, 71.1% of the respondents routinely checked their body temperature, 44.1% regularly disinfected doorknobs and cellphones, and 68.2% tried to maintain social distancing. Regarding behavioral changes in response to COVID-19, behavioral self-restraint, body-temperature checks, disinfection, and social distancing were considered positive behaviors for maintaining health, but holding off on being examined by a physician was considered negative behavior (reversal factor), with the scores converted to a 10-point scale.

The average score for the respondents was 6.67 ± 1.50; Cronbach’s α=0.662. Tables 5 and 6 indicate the analysis results, with positive behaviors and attitudes as the target variables and health literacy, age, and gender as the predictive variables.

**Table 5:** Univariate linear regression analysis of preventive action, predictive factors.

	Non-standardized coefficient		P-value	B 95% confidence interval	
	B	Standard error		Lower bound	Upper bound
(Constant values)	7.392	0.156	0.000	7.085	7.699
Under care of physician	-0.462	0.095	0.000**	-0.648	-0.275
(Constant values)	6.328	0.147	0.000	6.040	6.616
Gender	0.275	0.113	0.015*	0.054	0.496
(Constant values)	6.703	0.105	0.000	6.498	6.909
Employment	-0.013	0.033	0.696	-0.078	0.052
(Constant values)	6.664	0.103	0.000	6.460	6.867
Employment type	0.003	0.070	0.970	-0.135	0.140
(Constant values)	5.747	0.213	0.000	5.330	6.165
Health literacy	0.027	0.006	0.000**	0.015	0.040
(Constant values)	30.819	1.117	0.000	28.627	33.010
Age	0.569	0.231	0.014*	0.116	1.021
(Constant values)	6.850	0.142	0.000	6.571	7.129
Knowledge	-0.137	0.100	0.172	-0.334	0.060

*p<0.05

**p<0.01.

**Table 6:** Multivariate linear regression analysis of preventive action, predictive factors.

	Non-standardized coefficient		P-value	95% confidence interval	
	B	Standard error		Lower bound	Upper bound
(Constant values)	6.112	0.313	0.000	5.498	6.726
Under care of physician	-0.460	0.097	0.000**	-0.650	-0.271
Gender	0.343	0.116	0.003**	0.116	0.570
Employment	-0.005	0.034	0.886	-0.071	0.062
Employment type	-0.022	0.071	0.759	-0.161	0.118
Health literacy	0.027	0.006	0.000**	0.015	0.039

**p<0.01.

Multiple R-squared: 0.04868, Adjusted R-squared: 0.0439.

An association was observed between positive behaviors and age (the older the respondent, the more positive the behaviors), gender, physician care status, and health literacy. Care must be taken when predicting the positive attitudes and actions regarding COVID-19 prevention using this linear regression model, since the goodness of fit of this model is weak.

### (5) Intent (willingness) to undergo vaccination and reason for decision

35.8% of the respondents demonstrated an intent to undergo the coronavirus vaccination immediately upon availability, 27.1% preferred to vaccinate six months after availability, 25.7% preferred to vaccinate one year after availability, 3.6% preferred to vaccinate two years after availability, and 17.8% did not indicate an intent to undergo vaccination. Responses to indicating a desire to undergo vaccination were defined as vaccination willingness. Tables 7 and 8 indicate univariate and multivariate analyses, with vaccination acceptability as the target variable and gender, age, academic background, whether under a physician’s care, and health literacy as predictive variables.

**Table 7:** Univariate linear regression analysis of willingness to undergo vaccination, predictive factors.

	Non-standardized coefficient		P-value	B 95% confidence interval	
	B	Standard error		Lower bound	Upper bound
(Constant values)	4.249	0.140	0.000	3.975	4.524
Gender	-0.532	0.107	0.000**	-0.743	-0.321
(Constant values)	3.002	0.212	0.000	2.585	3.418
Age	0.125	0.044	0.004**	0.039	0.211
(Constant values)	4.051	0.152	0.000	3.753	4.348
Under care of physician	-0.290	0.092	0.002**	-0.471	-0.109
(Constant values)	2.837	0.206	0.000	2.434	3.240
Health literacy-50	0.023	0.006	0.000**	0.011	0.034

**p<0.01.

**Table 8:** Multivariate linear regression analysis of willingness to undergo vaccination, predictive factors.

	Non-standardized coefficient		P-value	95% confidence interval	
	B	Standard error		Lower bound	Upper bound
(Constant values)	3.681	0.395	0.000**	2.906	4.457
Gender	-0.491	0.111	0.000**	-0.708	-0.274
Age	0.032	0.046	0.493	-0.059	0.123
Under care of physician	-0.223	0.094	0.018*	-0.407	-0.039
Health literacy-50	0.021	0.006	0.000**	0.010	0.033

*p<0.05

**p<0.01.

Respondents who are male with a history of being under a physician’s care and higher health literacy had a significantly higher willingness to undergo vaccination. Reasons given by respondents for wanting to receive the vaccination immediately were because the benefits outweighed the risks as their work was in the service industry, in they would be better off vaccinated. Other responses included that they did not want to spread the virus to others, vaccines are safe, undergoing vaccination would ensure that the coronavirus does not spread, risk of infection would decrease, they worked with large numbers of people, undergoing vaccination would be better than doing nothing, older people undergoing the vaccination would allow for the observation of side effects, and that older people were at higher risk of becoming critically ill if they were infected.

In contrast, respondents gave the following responses regarding the reasons they would not undergo the vaccination: because they doubted the effectiveness of the vaccine, they had fears about the body’s reaction to the vaccines, they did not feel the need, they doubted the clinical trial results since the vaccines were developed so quickly, they have never even had the influenza vaccination, natural immunity from the spread of the coronavirus would be sufficient given that the vaccines are not a treatment, and they did not want to inject their body with something unknown.

### (6) Health and medical information sources and reliability

**Fig 1:**
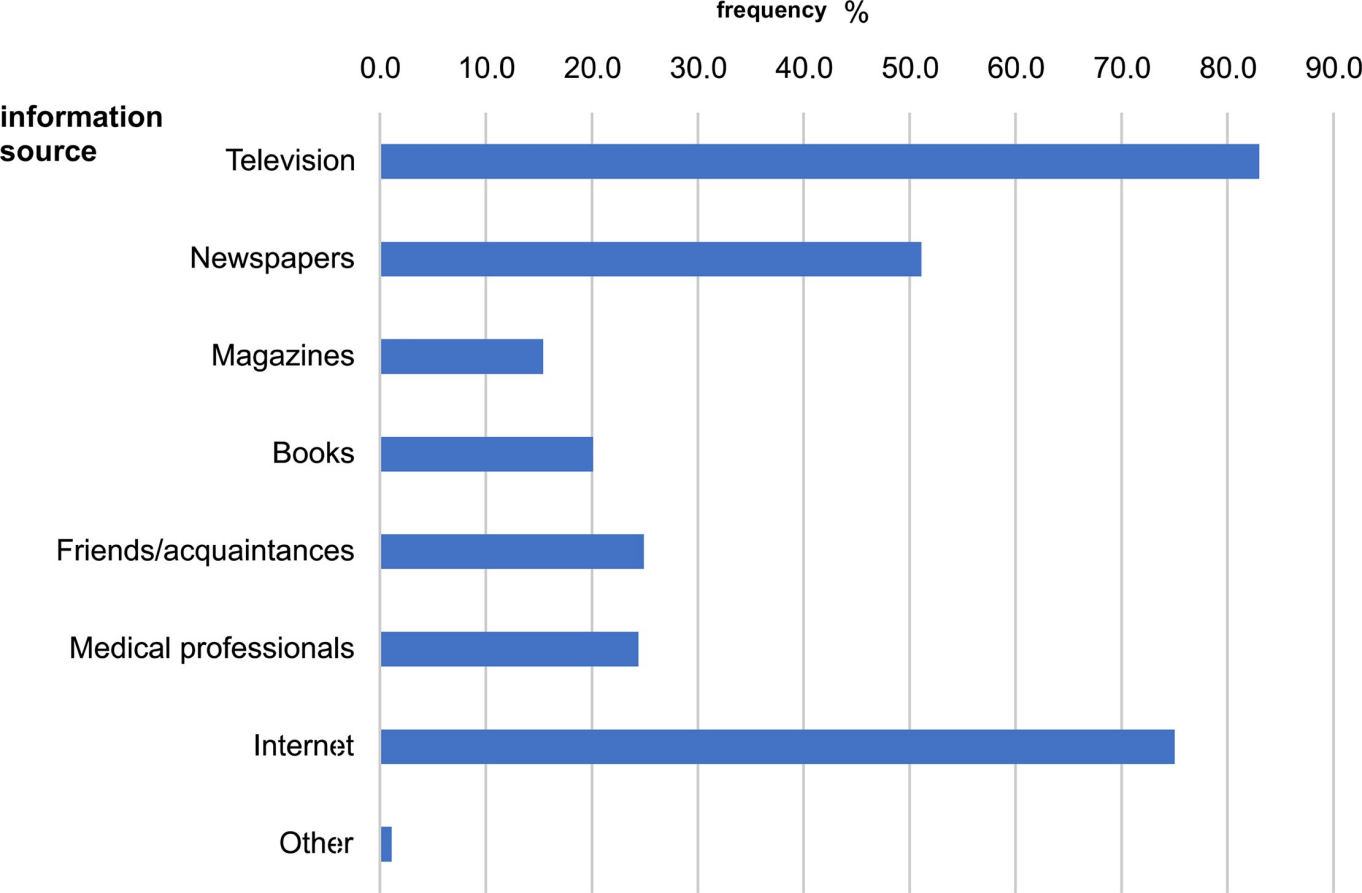
Sources of health and medical information.

Fig 1 shows the sources of health and medical information for educators. Television accounted for 83% of the total; newspapers, 51%; magazines, 15.4%; books and publications, 20%; friends/acquaintances, 24.3%; medical personnel, 24.4%; and the internet, 75%.

Table 9 indicates the reliability of the information (on a 5-point Likert scale). National government announcements measured at 2.57 ± 1.24; announcement by local government leaders: 3.11 ± 1.07; newspapers: 3.33 ± 0.95; television: 3.00 ± 1.04; family physician/pharmacy: 3.74 ± 0.78; friends and acquaintances: 2.95 ± 0.79; the Internet: 2.75 ± 0.85; and corona-related books and magazines: 3.29 ± 0.78. The reliability of information released by local governments was significantly higher than that released by the national government (p < 0.01).

**Table 9:** Reliability of information.

	Govt. Announcement	Local govt. leader announcement	Newspaper	TV	Family doctor, pharmacy	Friends, acquaintances	Internet	Coronavirus -related publications
Average value	2.57	3.11	3.33	3.00	3.74	2.95	2.75	3.29
Standard deviation	1.235	1.073	0.950	1.043	0.775	0.789	0.847	0.781

Table 10 indicates the analysis results, with willingness to undergo vaccination as the target variables and the reliability of the information as the predictive variables.

**Table 10:** Univariate linear regression analysis of willingness to undergo vaccination and information source reliability.

	Non-standardized coefficient		P-value	B 95% confidence interval	
	B	Standard error		Lower bound	Upper bound
(Constant values)	3.402	0.105	0.000	3.195	3.609
Govt. Announcement	0.075	0.037	0.042*	0.003	0.148
(Constant values)	3.035	0.139	0.000	2.761	3.308
Local govt. leader announcement	0.180	0.042	0.000**	0.097	0.264
(Constant values)	3.017	0.166	0.000	2.692	3.343
Newspaper	0.174	0.048	0.000**	0.080	0.268
(Constant values)	3.263	0.139	0.000	2.990	3.536
TV	0.111	0.044	0.012*	0.025	0.197
(Constant values)	2.624	0.224	0.000	2.185	3.062
Family doctor, pharmacy	0.260	0.059	0.000**	0.145	0.375
(Constant values)	3.590	0.177	0.000	3.241	3.938
Friends, acquaintances	0.002	0.058	0.975	-0.112	0.116
(Constant values)	3.579	0.156	0.000	3.273	3.885
Internet	0.006	0.054	0.915	-0.100	0.112
(Constant values)	3.087	0.198	0.000	2.698	3.475
Coronavirus -related publications	0.154	0.059	0.008**	0.040	0.269

**p<0.01.

The association between the willingness to receive the vaccine and the reliability of information from health care workers, announcements by national government and local government, newspaper articles, television, and books was recognized. On the other hand, no association was found between the reliability of friends’ information, the reliability of Internet information, and the desire to receive a vaccine.

## Discussion

Following initial reports of the COVID-19 outbreak in Wuhan, China in December 2019, the infection started spreading rapidly in February 2020. As of March 4, 2021, 114,853,685 confirmed cases and 2,554,694 deaths have been reported [40]. In addition, it has become clear that risks differ depending on individual factors such as age and the presence of underlying diseases [41, 42]. Initially, schools were locked down in many countries. In Japan, under the guidance from the Ministry of Education, Culture, Sports, Science and Technology, a hybrid of online and in-person classes were still being conducted because of the lower risk of the virus in younger people compared to the elderly and to avoid jeopardizing educational outcomes, with a policy in place to increase the rate of in-person lessons until that figure reaches 70% [43]. However, sporadic cluster outbreaks have been reported in schools [44], which suggests that educators have a key role in preventing the spread of the infection in educational settings is still significant.

Based on a survey of Japanese educators during the so-called third wave of viral infection spread, we noted that 90% of respondents refrained from going out and restricted eating out, but only a relatively low 68% worked to maintain social distancing from other people. Almost all respondents understood that contact or droplet infection (99.9%) was the cause of the virus spread, but only 70% of the respondents took it upon themselves to check their body temperature and 40% to disinfect cellphones and doorknobs, suggesting that there was a slight discrepancy between the understanding of infectious diseases and preventive actions. In addition, information from medical professionals was scored as being the source of the highest reliability, with the reliability of announcements by the national government the lowest among other information sources.

This study showed that the health literacy of Japanese educators is higher than that of the general Japanese public’s health literacy as shown in previous studies by 25.3 points [38]. Health literacy is a self-assessment of the reliability and understanding of medical information and is therefore considered to be highly associated with feelings of self-affirmation [45].

In this study, a level of association between academic background, highest educational attainment, and age were observed in health literacy. The study suggested that age and university degree might be more strongly linked to health literacy and feelings of self-affirmation compared to academic background. Preventive actions taken by educators against the coronavirus appeared to be associated with health literacy, age, gender, and whether respondents were under a physician’s care during the prolonged period of COVID-19 induced lifestyles when the third wave of viral infections spread in Japan.

In this study, 35% of the participants avoided visiting medical or dental clinics, which was nearly identical to the Cameroon survey results. Existing studies were conducted during the period when COVID-19 was first confirmed and spread, and thus were not conducted in situations in which numbers of infected individuals rose and fell repeatedly [22–24, 32–34, 46]. Some respondents who indicated that they could not maintain social distancing may have been educators who were unable to do so given the nature of their work. By having educational institutions implement facility improvements and adopt other precautionary measures for the appropriate management of infectious diseases, students and faculties can be protected and the educational opportunities of students can be secured.

Several studies have reported on public attitudes and knowledge of COVID-19 in the early stages of the spread of the virus during March–May 2020 [22–24, 34, 47]. Studies in the United Kingdom and the United States conducted in the early stages of transmission have reported that while knowledge of the transmission routes of COVID-19 was high, awareness of behavior to prevent infection was inadequate [48]. Previous studies have reported that knowledge and attitudes about COVID-19 and practices of preventive actions are associated with age, vocation, education level, location of residence, and gender differences [22–24, 32–34, 48, 49].

In a survey conducted in Cameroon from April to May 2020, when the virus was spreading rapidly, 84% of respondents were aware of the routes of infection and how the spread of the infection occurred. The Cameroon study, which assessed attitudes that led to ongoing health examinations and testing for coronavirus infection, reported that 69% of study participants had a positive attitude [50]. According to a Canadian study, while Canadian respondents accessed television and newspapers as sources of information at a high rate of 75%, 60% of respondents indicated that government announcements and domestic media coverage provided the most reliable information [51]. In our study, there was a connection between the willingness to vaccinate and health literacy, as well as announcements from national and local governments, newspaper/television coverage, books, and articles. On the other hand, the reliability of SNS information transmitted on the Internet was low, and there was no observable association with the intention to vaccinate.

Therefore, we suggest that to increase the vaccination rate, it is necessary to improve the health literacy of individuals and at the same time increase the credibility of government coverage and media.

Trust in the national government can lead to more preventive behavior and control over COVID-19 [51, 52]. According to the results of this study, information from medical professionals is the most reliable for educators. The reliability of information from regional and/or local governments, newspapers, magazines, and from friends and others is about the same. It became clear that the reliability of the internet was relatively lower, and the information of the central government was the lowest. In order to improve awareness in the field of education and control COVID-19 in Japan, it is necessary to increase sustainable behavioral change and trust in the central government’s public relations.

## Conclusion

This study revealed that age, gender, whether a respondent was under a physician’s care, and health literacy affected certain behaviors of educators, including willingness to undergo vaccination, and take preventive actions. Increasing the health literacy of educators might have a positive effect on behavioral changes not only for educators but also for students. There are two ways to promote preventive action, including vaccination. One is improving the educator’s own health literacy, and the other is implementing training activities for women and young educators who have never visited a hospital. Since the relationship between the information sent by medical staff and local governments and individual vaccination willingness has been confirmed, it is possible to increase willingness to undergo vaccination, and by default, increase the vaccination rate, by increasing the reliability of medical information. With respect to the reliability of medical information, this study suggested that the reliability of national government public relations efforts is lower than the reliability of regional/local government public relation efforts, as well as information from mass media. For this reason, it is necessary to reexamine how information is disseminated by the national government and increase the degree of trust in that information among the public. The study suggests that it is important to improve the provision of appropriate information and health literacy for educators not only during times of pandemic outbreaks, but also when restrained lifestyles of the public is prolonged.

### Strengths and limitations of this study

This study is the first to investigate the knowledge, attitude, and intention of vaccination of educators related to COVID-19. Since educators are key persons in ensuring the quality of education and controlling infections in the field of education, such research is useful in considering risk management in the field of education and infection control strategies in the event of a new infectious disease. In addition, this study was conducted during the occurrence of the third wave of infection, when the wave of infection was rapidly spreading, which is different from the initial stage of infection spread, during which continuous behavioral restriction and prevention was required. It can also contribute to improved risk communication for voluntarily implementing actions.

However, this study has some limitations. First, since it was an Internet survey, there may be a selection bias as only Internet users could participate in this study. In addition, the intention of vaccination may change depending on the status of infection, efficacy, and confirmation of safety. Therefore, further research is needed on an ongoing basis.

## Supporting information

S1 Data (XLSX)
